# Curcumin and Curcumol Inhibit NF-*κ*B and TGF-*β*_1_/Smads Signaling Pathways in CSE-Treated RAW246.7 Cells

**DOI:** 10.1155/2019/3035125

**Published:** 2019-03-17

**Authors:** Ning Li, Tian-Hao Liu, Jing-Ze Yu, Chen-Xi Li, Yang Liu, Yue-Ying Wu, Zhong-Shan Yang, Jia-Li Yuan

**Affiliations:** ^1^Faculty of Basic Medical Sciences, Yunnan University of Chinese Medicine, Yuhua Road, No. 1024, Kunming 650500, Yunnan, China; ^2^Chinese Medicine College, Jinan University, 601 Huangpu West Avenue, Guangzhou 510632, Guangdong, China

## Abstract

E-Zhu (*Curcuma zedoaria*) is known as a classical traditional Chinese medicine and widely used in the treatment of cancers, cardiovascular disease, inflammation, and other diseases. Its main components include curcumol and curcumin, which have anti-inflammatory and antifibrosis effects. Here we established an* in vitro* inflammatory injury model by stimulating RAW246.7 cells with cigarette smoke extract (CSE) and detected the intervention effects of curcumin and curcumol on CSE-treated Raw246.7 macrophage cells to explore whether the two compounds inhibited the expression of inflammatory cytokines by inhibiting the NF-*κ*B signaling pathway. We detected the antifibrosis effects of curcumin and curcumol via TGF-*β*_1_/Smads signaling pathways. The model of macrophage damage group was established by CSE stimulation. Curcumol and curcumin were administered to Raw246.7 macrophage cells. The efficacy of curcumol and curcumin was evaluated by comparing the activation of proinflammatory factors, profibrotic factors, and NF-*κ*B and TGF-*β*_1_/Smads signaling pathway. In addition, CSE-treated group was employed to detect whether the efficacy of curcumol and curcumin was dependent on the NF-*κ*B signaling via the pretreatment with the inhibitor of NF-*κ*B. Our findings demonstrated that curcumol and curcumin could reduce the release of intracellular ROS from macrophages, inhibit the NF-*κ*B signaling pathway, and downregulate the release of proinflammatory factor. Curcumol and curcumin inhibited the TGF-*β*_1_/Smads signaling pathway and downregulated the release of fibrotic factors. Curcumin showed no anti-inflammatory effect in CSE-treated cells after the inhibition of NF-*κ*B. Curcumol and curcumin showed an anti-inflammatory effect by inhibiting the NF-*κ*B signaling pathway.

## 1. Introduction

Chronic obstructive pulmonary disease (COPD) is a chronic respiratory disease which seriously endangers human health. According to the study on Chinese Lung Health [1], the number of COPD patients in China is about one hundred million and the COPD incidence is up to 13.7% in adults 40 years old and above [2]. Chronic airway inflammation and airway remodeling are the basic pathological features of COPD. Chronic inflammation is important in the development of COPD and the decline of lung function [3]. NF-*κ*B (nuclear transcription factor kappa B) plays an important role in the development of COPD [4]. NF-*κ*B signaling pathway is an important signal transduction pathway in inflammatory diseases and can regulate the secretion of various inflammatory mediators. As one of the most important phagocytes in human body, macrophages release a series of inflammatory mediators such as TNF-*α* (tumor necrosis factor-*α*), IL-6 (Interleukin-6), IL-1*β* (Interleukin-1*β*) [5], and ROS (active oxygen cluster) and activate NF-*κ*B, thus increasing the inflammatory response and exacerbating airway inflammation and severe airway damage [6]. The aggravation of airway inflammation leads to the injury and repeated hyperplasia of the airway wall, which results in the deposition of connective tissues in airway wall, the thickening of smooth muscle of airway wall, and the final remodeling of airway [7]. *α*-SMA (alpha-smooth muscle actin), a characteristic marker of smooth muscle, can reflect the changes in the number and contractility of smooth muscle. It is the structural basis of the contraction of smooth muscle cells. TGF-*β*_1_ (transforming growth factor beta1) signaling pathway via the phosphorylation of Smads protein can activate the expression of airway smooth muscle cell protein *α*-SMA and promote airway remodeling and fibrosis. Therefore, the inhibition of TGF-*β*1/Smads signaling pathway can reduce the expression of *α*-SMA and the deposition of extracellular matrix and alleviate airway fibrosis [8]. The traditional Chinese medicine E-Zhu is also known as* Curcuma zedoaria*, which is the dry rhizome of the ginger plants [9]. Modern chemical and pharmacological studies show that rhizomes of* Curcuma zedoaria* turmeric mainly contain* Curcuma*, volatile oils, polysaccharide, and alkaloids. The volatile oils include curcumol, *β*-elemene, and curdione. In these compounds, curcumol and curcumin are the most widely used medical components and play an important role in anti-inflammatory [10], antitumor [11], antioxidant [12], and antithrombosis activities [13]. In order to explore the molecular mechanism of curcumol and curcumin, the main components of the traditional Chinese medicine E-Zhu, in the treatment of COPD, in this experiment, macrophage Raw246.7 cells, were stimulated by CSE (cigarette smoke extract) and the damage model of COPD was simulated in vitro to observe whether curcumol and curcumin could reduce the expressions of inflammatory cytokines and fibrotic factors in macrophages by inhibiting the NF-*κ*B and TGF-*β*_1_/Smads signaling pathways.

## 2. Materials and Methods

### 2.1. Cell Culture and CSE Preparation

Mouse macrophage cells Raw246.7 provided by Professor Cheng-gang Zou (Yunnan University, China) were grown in DMEM medium containing 10% FBS and 1% penicillin-streptomycin and maintained at 37°C in the environment containing 5% CO_2_. To determine whether curcumol and curcumin affected the production of proinflammatory cytokines via NF-*κ*B signal pathway, 1 *μ*M Bay 11-7082 as an inhibitor of NF-*κ*B signal pathway was added to the medium. After incubation for 24 h, the cells were harvested for further study.

According to the previous method with minor modifications [14], a lighted cigarette was connected to a peristaltic pump apparatus and cigarette smoke was collected until the cigarette was burned. Then smoke was injected into the blue cap bottle containing the 25 mL of DMEM medium and dissolved in the medium. The pH value was adjusted to 7. In this way, the CSE solution was obtained. Fetal bovine serum was added according to 10% volume to the CSE solution and filtered with 0.22 *μ*m filter membrane to remove bacteria. Finally, the DMEM medium containing CSE was prepared and sealed at 4°C.

### 2.2. Reagents and Instruments

Curcumol, curcumin (Chengdu Manste Biotechnology Co., Ltd.), DMEM high sugar culture liquid [Thermo Fisher biochemical products (Beijing) Co., Ltd.], fetal bovine serum (FBS) (Biological Industries), CellTiter 96 AQueous One Solution Cell Proliferation Assay (MTS), Red Tashan cigarette (Hongta Tobacco Co., Ltd.), SYBR Premix EX Tap II fluorescent quantitative reagent box (Takara Biological Engineering Co., Ltd.), Anti-Phospho-NF-*κ*B p65 antibody (Cell Signaling Technology Company), Anti-Smad2+Smad3 (phosghoT8) antibody (Abcam Company), NF-*κ*B inhibitor Bay 11-7082 (Beyotime Biotech Company), DCFH-DA (Sigma Company), and BCA protein concentration assay kit (Beyotime Biotech Company) were used in the study.

### 2.3. Suitable Concentrations of Two Compounds Screened by MTS and Caspase-3 Apoptosis Assays

Raw246.7 macrophage cells in the logarithmic growth phase were inoculated in 96-well plates (100 *μ*L per well). The cells were divided into curcumol group and curcumin group. The drugs were added to wells according to different concentrations (1, 5, 10, 50, 100, and 200 g/mL) and the normal control group and zeroing well were arranged. Except the zeroing well, 3 pairs of vice wells were arranged in each group and placed in the cell culture box containing 5% CO_2_ at 37°C. After 12 h, 96-well plates were taken out. The thawed MTS reagent was added to 96-well plates at a dose of 20 *μ*L per well. After incubation for 4 h, the absorbance at 490 nm in each well was detected on the enzyme labelling instrument. Raw246.7 macrophage cells were collected in the logarithmic growth phase. After 15 min ice bath cracking and centrifugation for 10-15 min (4°C, 16000-20000 g), the supernatant was collected for the detection of the enzyme activity of caspase-3. After incubation at 37°C for 60-120 min, the absorbance at 405 nm was detected when the color turned yellow.

### 2.4. Detection of ROS Fluorescence Expression in Cells by DCF Staining

The Raw246.7 macrophage cells in the logarithmic growth phase were digested and prepared into a cell suspension, which was evenly added into six-well plates containing glass slides for cell growth. The cells were grouped. After curcumol and curcumin were added, DCF was added (10 *μ*M per well). After 20 min incubation in dark and washing with PBS for three times, the fluorescence intensity of ROS was observed with a fluorescence microscope under 488 nm excitation wave and 525 nm emission wave. After the observation, the lysed cells were mixed for 10 min in a shaker at 37°C and the fluorescence intensity under 488 nm excitation wave and 525 nm emission wave were detected by the enzyme labelling instrument.

### 2.5. Quantitative Real Time PCR

Total RNA was extracted according to the instructions of the kit. Then the mRNA was reversely transcribed into cDNA according to the operation procedure: denaturation for 30 s at 95°C, 40 amplification cycles of 15 s at 95°C, 60 s at 60°C, and 15 s at 95°C, and extension for 34 s at 60°C. The quantitative gene expression was tested by 2^△△Ct^ method. The primers used for PCR were provided in Table 1.

### 2.6. Western Blotting

Total protein lysates were estimated by the BCA protein assay and the standard curve of proteins was plotted. The lysates of total protein per well were separated with 10% SDS polyacrylamide gel and then transferred onto PVDF membrane. Membranes were used to detect the phosphorylated forms of the proteins. Primary antibodies were Anti-Phospho-NF-*κ*B p65 (1: 3000 dilution; Cell Signaling Technology), Anti-Smad2+Smad3 antibody (1: 1000 dilution; Abcam Company), and anti-beta Actin Monoclonal Antibody (1:3000 dilution; Proteintech Group). The secondary antibody was a peroxidase-coupled anti-mouse or rabbit IgG (1:4000 dilution; Biosharp Company). The membrane was exposed to X-ray photography dark box. The film was developed by ECL protein imprinting substrate method. Gray levels were tested by ImageJ software.

### 2.7. Statistics and Analysis

The one-way analysis of variance was performed in SPSS 18.0 software. The results followed a normal distribution. Data were expressed as mean ± standard deviation.* P*<0.05 was considered to be statistically significant.

## 3. Results

### 3.1. Effects of Curcumol and Curcumin on the Viability of Raw246.7 Macrophage Cells

**Figure 1:**
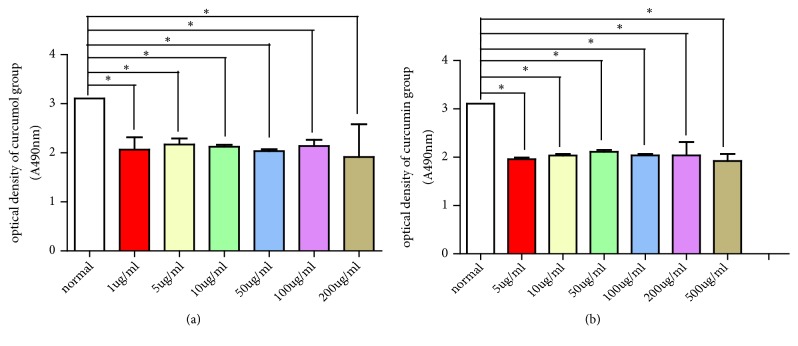
(a) Effects different concentrations of curcumol (a) and curcumin (b) on the viability of Raw246.7 macrophage cells.

Partial mother liquids of curcumol and curcumin were diluted with DMSO to different concentrations (1, 5, 10, 50, 100, 200, and 500 g/mL). The viability of Raw246.7 cells was detected under different concentrations of curcumol and curcumin. In curcumin group, the cell viability under a curcumol concentration of 5 *μ*g/mL was significantly high (*P*<0.05). In curcumin group, the cell viability under a curcumol concentration of 50 *μ*g/mL was significantly high when it was found ((*P* < 0.05, Table 2 and Figure 1). The cell growth state was observed under 5 *μ*g/mL curcumol and 50 *μ*g/mL curcumin.

**Figure 2:**
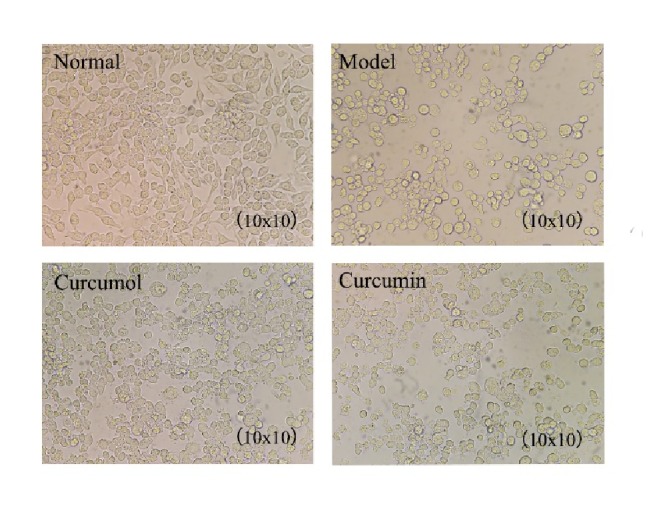
Growth state of Raw246.7 macrophage cells.

In normal group, the cells were small, spindle-shaped, and in good growth condition without obvious dead cells. In model group, after stimulation with CSE, the cells were spherical, decreased, and aggregated into clusters with floating cell debris. In the curcumol and curcumin groups, the cell growth density increased compared to model group and the death rate of cells decreased (Figure 2).

**Figure 3:**
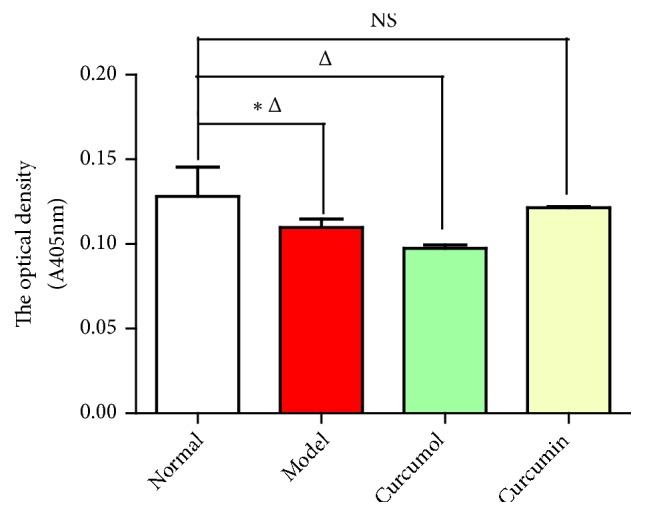
Effects of curcumol and curcumin on the level of caspase-3. Data are expressed as mean ± SD of three independent experiments. ^△^*P*<0.05 (comparison with normal group); ^⋆^*P*<0.05 (comparison with model group); NS, not significant versus control.

The caspase-3 apoptosis detection method was used to detect the apoptosis level of the cells. The apoptosis level of normal group was close to that of model group. The apoptosis level of curcumol group was close to that of normal group. The apoptotic level of curcumin group was close to that of other groups. The differences in the apoptosis levels in different groups were similar, indicating that 5 *μ*g/mL curcumol and 50 *μ*g/mL curcumin had little toxicity on cells (Figure 3 and Table 3).

### 3.2. Curcumol and Curcumin Reduced the Release of ROS and Downregulated the Relative mRNA Levels of Inflammatory Factors and the Expression Level of P- NF-*κ*B p65 Protein

**Figure 4:**
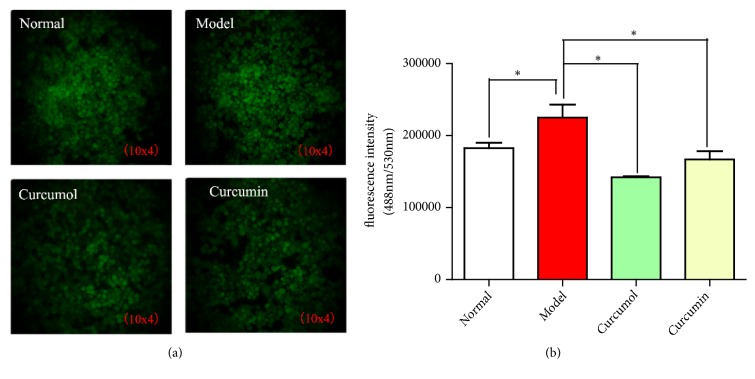
(a) Effects of curcumol and curcumin on fluorescence intensity of macrophage ROS. (b) Effects of curcumol and curcumin on intracellular ROS. Data are expressed as mean ± SD of three independent experiments.

Compared with normal group, model group showed the increased expression of fluorescence, indicating that a large number of ROS was produced in the cells. CSE could induce intracellular ROS release and produce inflammatory reaction. Compared with model group, the curcurol and curcumin groups showed the decreased fluorescence intensity. Curcumol group had the lowest fluorescence intensity (*P*< 0.05). Curcumol and curcumin could reduce the release of ROS in cells and the effect of curcumae on reducing the release of ROS was more significant (Figure 4).

**Figure 5:**
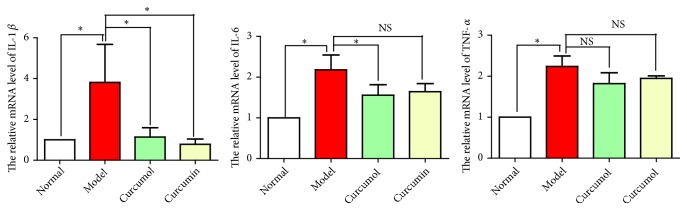
Effects of curcumol and curcumin on the relative mRNA levels of IL-1*β*, IL-6, and TNF-*α*. Data are expressed as mean ± SD of three independent experiments.

The expression levels of inflammatory factors in model group were high, indicating that CSE stimulated macrophages to release a large number of inflammatory factors. Curcumol and curcumin could reduce the expressions of inflammatory factors to varying degrees. The effect of curcumol on reducing the expressions of inflammatory factors was more significant (Figure 5).

**Figure 6:**
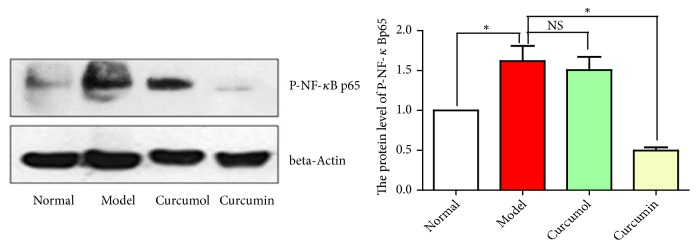
Effects of curcumin and curcumol on the protein level of P-NF-*κ*Bp65. Data are expressed as mean ± SD of three independent experiments.

Compared with normal group, model group showed the significantly increased expression level of P-NF-*κ*B p65 protein, indicating that CSE activated the NF-*κ*B signaling pathway and released a large number of inflammatory factors. Compared with model group, the curcumol and curcumin groups showed the decreased expression level of P-NF-*κ*B p65 protein and the effect in curcumin group was more significant (*P*<0.05). In summary, curcumol and curcumin could inhibit the NF-*κ*B signaling pathway and reduce the expressions of inflammatory factors (Figure 6).

### 3.3. Effects of Curcumol and Curcumin on the Relative mRNA Levels of Inflammatory Factors after Inhibiting NF-*κ*B

**Figure 7:**
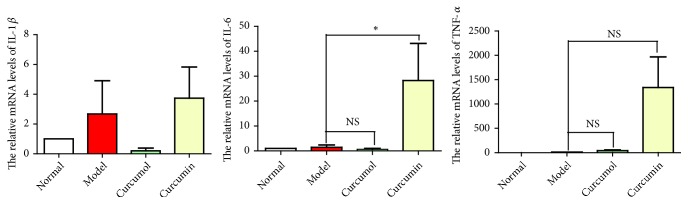
Effects of curcumol and curcumin on the relative mRNA levels of IL-1*β*, IL-6, and TNF-*α* after inhibiting NF-*κ*B. Data are expressed as mean ± SD of three independent experiments.

After inhibiting NF-*κ*B, the expressions of inflammatory factors in curcumin group increased and the effect of relieving inflammation decreased. However, the expression levels of inflammatory factors IL-1*β* and IL-6 in curcumol group decreased and the expression level of TNF-*α* increased. Therefore, we speculated that curcumin could inhibit IL-6, IL-1*β*, and TNF-*α* via NF-*κ*B signal transduction pathway to alleviate the inflammatory reaction. Curcumol alleviated the inflammatory response by inhibiting TNF-*α* through the NF-*κ*B signaling pathway (Figure 7).

### 3.4. Curcumol and Curcumin Reduced the Release of Fibrotic Factors and the Level of Phosphorylation Smad2/3

**Figure 8:**
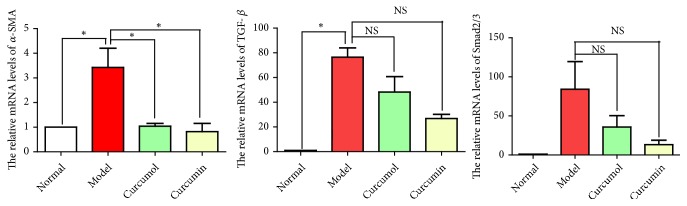
Effects of curcumol and curcumin on the relative mRNA levels of *α*-SMA, TGF-*β*, and Smad2/3. Data are expressed as mean ± SD of three independent experiments.

Compared with normal group, model group showed the increased relative mRNA levels of the *α*-SMA, TGF-*β*, and Smad2/3, indicating that CSE stimulated macrophages to secrete a large number of cytokines causing fibrosis. Compared with model group, the curcumol and curcumin groups showed the decreased relative mRNA levels of *α*-SMA, TGF-*β*, and Smad2/3, indicating that curcumol and curcumin downregulated the expressions of fibrotic factors (Figure 8).

**Figure 9:**
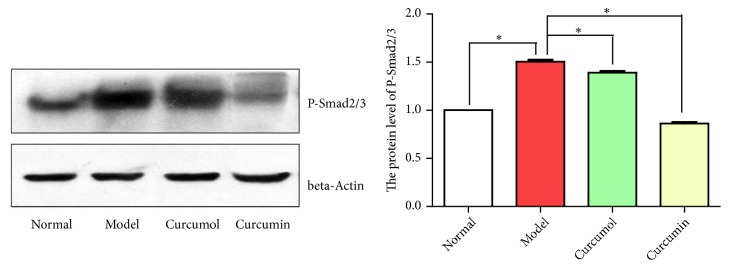
Protein level of P-Smad2/3 of curcumin and curcumol. Data are expressed as mean ± SD of three independent experiments.

Compared with normal group, model group showed the increased expression level of P-Smad2/3. Compared with model group, the curcumol and curcumin groups showed the significantly decreased expression level of P-Smad2/3 (*P*<0.05), indicating that curcumin and curcumin downregulated the expression of the fibrotic factor via the TGF-*β*_1_/Smads signaling pathway (Figure 9).

### 3.5. Effects of Curcumol and Curcumin on the Relative mRNA Level of *α*-SMA and the Expression Level of P-Smad2/3 after Inhibiting NF-*κ*B

**Figure 10:**
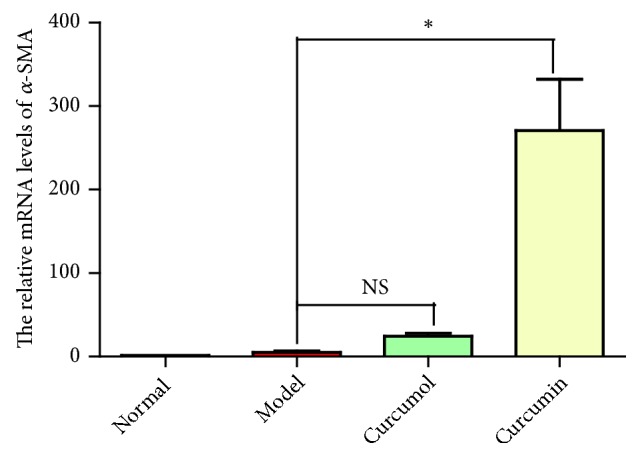
Effects of curcumol and curcumin on the relative mRNA levels of *α*-SMA after inhibiting NF-*κ*B. Data are expressed as mean ± SD of three independent experiments.

**Figure 11:**
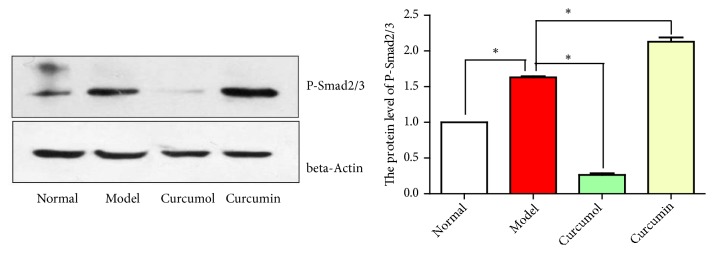
Effects of curcumin and curcumol on the protein level of P-Smad2/3 of after inhibiting NF-*κ*B. Data are expressed as mean ± SD of three independent experiments.

After inhibiting NF-*κ*B, the relative mRNA level of *α*-SMA and Smad2/3 protein phosphorylation level in curcumin group increased significantly (*P*<0.05, Figures 10 and 11). Curcumin could alleviate fibrosis after inhibiting NF-*κ*B.

## 4. Discussion

The main chemical components of traditional Chinese medicine E-Zhu include two major categories: volatile oil (curcumol) and curcumin. Curcumin has long been used in antiviral, antioxidant, and anti-inflammatory applications and the management of other pharmacological activities [15–19]. In addition, curcumol shows the pharmacological activities, including anti-tumor, antivirus, antibacterial, and anti-thrombus activities [20–22]. In the study, curcumol and curcumin are used to interfere with macrophage injury model and explore its molecular mechanism in inflammation and fibrosis in this experiment.

NF-*κ*B signaling pathway and TGF-*β*1_1_/Smad signaling pathway are the key factors in the development of inflammation and fibrosis, and play an important role in the development of inflammatory diseases. NF-*κ*B is a widely distributed nuclear transcription factor activated by various stimuli and transported into the nucleus. The gene transcription is regulated by binding promoter or enhancer to DNA's *κ*B site under the action of cosuppression factor and coactivator factor. Curcumin inhibits dextran sulfate sodium-induced DNA binding of NF-*κ*B in mouse colon [23]. Curcumin regulates different proinflammatory and profibrotic cytokines and plays a crucial role in the mitigation of liver diseases and inrenal ischemia-reperfusion injury. Researchers demonstrated that the protective effect of curcumin inrenal ischemia-reperfusion injury is associated with suppressing NF-KB mediating inflammation; curcumin shows the inhibitory effect on HBV by activating the pathways suppressing the activation of NF-*Κ*b [24, 25]. In addition, after the activation of TGF-*β*_1_ superfamily receptor, the biological signal is transmitted to the Smads protein in the middle and lower streams of the cytoplasm. The activated TGF-*β*_1_ receptor phosphorylates Smad2 and Smad3, which are then transferred to the nucleus to participate in the transcription of related genes, thus inhibiting the degradation of the extracellular matrix and the synthesis of enzymes such as collagenase and matrix lysine and inducing collagen deposition [26]. We demonstrated that curcumol and curcumin reduced the release of ROS in Raw246.7 cell macrophage and lessened the effect of oxidative stress. Curcumin can reduce the expression levels of TNF-*α*, IL-6, and IL-1*β* by inhibiting the NF-*κ*B signaling pathway. Curcumol inhibits the expression of the inflammatory factor TNF-*α* by inhibiting the NF-*κ*B signaling pathway. Curcumin can inhibit the activation of NF-*κ*B, reduce the binding of P65 to DNA, and lower the levels of TNF-*α* and IL-1*β* in lung tissue and serum [27]. In the study, we provided the evidence that curcumol and curcumin showed an anti-inflammatory effect by inhibiting the NF-*κ*B signaling pathway. After the intervention of NF-*κ*B inhibitor Bay 11-7082, the expression level of the inflammatory factor TNF-*α* in curcumin group and curcumol group increased and the expression levels of inflammatory factors IL-1*β* and IL-6 in curcumin group also increased. The inhibition of PI3K/NF-*κ*B pathway by curcumol leads to the apoptosis of HSC-T6 (hepatic stellate cells) and curcumol is a potential candidate for further preclinical study on the treatment of liver fibrosis. In addition, studies investigated the effect of curcumol in suppressing TGF-*β*_1_ led to the decrease of epithelial-mesenchymal transition occurrence. Reports also show that curcumol may modulate inflammatory and enhanced angiogenesis, resulting in better wound healing [28–30]. Our findings demonstrated that curcumol and curcumin could inhibit the TGF-*β*1/Smads signaling pathway too by reducing the mRNA expression levels of fibrotic factors *α*-SMA, Smad2/3 and TGF-*β*.

In conclusion, our findings perfectly supported the hypothesis demonstrated that curcumol and curcumin could reduce the expressions of inflammatory factors by inhibiting the NF-*κ*B signaling pathway and TGF-*β*_1_/Smads signaling pathway.

## Figures and Tables

**Table 1:** Primers used in the experiments.

Name	Gene synthesis sequence number
IL-6	F: 5′-TCCATCCAGTTGCCTTCTTG-3′
	R: 5′-AAGCCTCCGACTTGTGAAGTG-3′
IL-1 *β*	F: 5′-CCAGGATGAGGACATGAGCA-3′
	R: 5′-CGGAGCCTGTAGTGCAGTTG-3′
TNF-*α*	F: 5′-ACTGGCAGAAGAGGCACTCC-3′
	R: 5′-GCCACAAGCAGGAATGAGAA-3′
*α*-SMA	F: 5′-GAGCATCCGACACTGCTGAC-3′
	R: 5′-GCACAGCCTGAATAGCCACA-3′
Smad2/3	F: 5′-GGAACCTGCATTCTGGTGTT -3′
	R: 5′-CGAGTTTGATGGGTCTGTGA -3′
TGF-*β*	F: 5′-AGCAACAATTCCTGGCGATACCTC-3′
	R: 5′-TCAACCACTGCCGCACAACTC-3′
Beta-actin	F: 5′-GATGACGATATCGCTGCGCTC-3′
	R: 5′-CTGACCCATACCCACCATCACAC-3′

**Table 2:** Effects of different concentrations of curcumol and curcumin on the cell viability of Raw246.7 macrophage cells.

Groups	Concentrations (*μ*g/mL)	Optical density	Groups	Concentrations (*μ*g/mL)	Optical density
Normal	-	3.11 ± 0.01	Normal	-	3.11 ± 0.01
	1	2.07 ± 0.25^⋆^		5	1.96 ± 0.04^⋆^
	5	2.17 ± 0.12^⋆^		10	2.04 ± 0.03^⋆^
Curcumol	10	2.13 ± 0.04^⋆^	Curcumin	50	2.11 ± 0.04^⋆^
	50	2.04 ± 0.06^⋆^		100	2.04 ± 0.07^⋆^
	100	2.14 ± 0.13^⋆^		200	2.04 ± 0.28^⋆^
	200	2.12 ± 0.34^⋆^		500	1.92 ± 0.15^⋆^

Data are expressed as mean ± SD of three independent experiments. ^⋆^*P*<0.05 was defined as statistically significant.

**Table 3:** Effects of curcumol and curcumin on the level of caspase-3.

Group	A_405_
	mean ± SD
Normal group	0.13 ± 0.02^⋆^
Model group	0.12 ± 0.01^△^
Curcumol group	0.10 ± 0.00^△^
Curcumin group	0.12 ± 0.00

Data are expressed as mean ± SD of three independent experiments. ^△^*P*<0.05 (comparison with normal group); ^⋆^*P*<0.05 (comparison with model group).

## Data Availability

The data used to support the findings of this study are included within the article and can be made freely available. Any questions of data will be considered to be answered by the corresponding author.
